# βKlotho is identified as a target for theranostics in non-small cell lung cancer

**DOI:** 10.7150/thno.35582

**Published:** 2019-10-12

**Authors:** Fan Li, Xiyao Li, Ziming Li, Wenxiang Ji, Shun Lu, Weiliang Xia

**Affiliations:** 1 State Key Laboratory of Oncogenes and Related Genes, Renji-Med X Clinical Stem Cell Research Center, Ren Ji Hospital, School of Medicine and School of Biomedical Engineering, Shanghai Jiao Tong University, Shanghai, China; 2 Shanghai Lung Cancer Center, Shanghai Chest Hospital, Shanghai Jiao Tong University, Shanghai, China

**Keywords:** βKlotho, non-small cell lung cancer, molecular target, apoptosis, theranostics

## Abstract

Non-small cell lung cancer (NSCLC) remains a great challenge, calling for the identification of novel molecular targets with diagnostic/therapeutic value. Here, we sought to characterize the expression of βKlotho and its anti-tumor roles in NSCLC.

**Methods:** The expression of βKlotho was examined in NSCLC cells and tissues by western blot, qRT-PCR and immunohistochemistry staining respectively. Biological roles of βKlotho were revealed by a series of functional *in vitro* and *in vivo* studies. Serum βKlotho concentrations of patients were measured using specific ELISA methods.

**Results:** Serum βKlotho concentrations of NSCLC patients were significantly lower than the control group. Moreover, βKlotho expression was negatively associated with lymph node metastasis, overall survival and progression-free survival. Overexpression of βKlotho or exogenous βKlotho administration inhibited the proliferation and migration of NSCLC cells, accompanied by induction of apoptosis, G1 to S phase arrest, and inactivation of ERK1/2, AKT and STAT3 signaling. Furthermore, βKlotho overexpression inhibited NSCLC tumor growth *in vivo*.

**Conclusions:** βKlotho serves as a novel target for theranostics in NSCLC, which has potential clinical applications in the future.

## Introduction

Approximately 25% of all cancer deaths result from lung cancer. Despite the steady decrease in smoking and advances in earlier diagnosis through screening with low-dose computed tomography, the 5-year survival rate is still merely 19%, partly because more than 50% of cases are diagnosed at an advanced stage 1. In China, the mortality rate of lung cancer for both males and females ranks top among all cancer types 2. Non-small cell lung cancer (NSCLC), of which lung adenocarcinoma (LADC) and lung squamous cell carcinoma (LSQ) are the most common subtypes, accounts for nearly 85% of lung cancer and the landscape of treatments has been changed by targeted therapies. NSCLC patients harboring genetic alterations in EGFR, ALK, ROS1 and BRAF can implement specific targeted therapies with superior efficacy and low toxicity. However most patients experience relapse within a few years 3. Uncovering the mechanisms that drive lung cancer progression and underlie treatment resistance is an unfailing quest for cancer biologists and clinicians.

The klotho family proteins are comprised of the founding member Klotho (or alpha klotho), beta and gamma klotho, and are recognized for their special roles in energy metabolism 4, aging, muscle regeneration 5 and Alzheimer disease 6. Klotho was originally identified as an anti-aging gene 7, as it can extend life span when overexpressed 8. Interestingly, Klotho has been shown to suppress a range of cancer types 9-12 and its expression is usually downregulated in cancer due to hypermethylation in the promoter region. Overexpression of Klotho has been found to inhibit tumor growth in various animal models through inhibition of the TGFβ1, WNT, FGF2, and IGF1 signaling pathways 9-14. Indeed, in small cell lung cancer, klotho predicts good clinical outcome 15.

βKlotho (KLB), however, plays a more complex role in cancers based on current literacy. The KLB gene also encodes the single pass transmembrane protein 16 that serves as a catalytic subunit to mediate intracellular signaling. Crystal structures of Klotho and KLB have been revealed and KLB shares 41% similarity in amino acid sequence with Klotho 16-18. KLB was reported to act as a tumor promotor in human bladder cancer 19, and elevated KLB expression was detected in hepatocellular carcinoma tissues to provide an oncogenic role 20, 21. Contrarily, KLB has also been demonstrated to suppress tumor growth in hepatocellular carcinoma by regulating Akt/GSK-3β/Cyclin D1 signaling pathway 22, 23 and KLB level was significantly decreased and associated with fibrosis stage in liver tissue 24. Hence, KLB appears to play either tumor-promoting or tumor-suppressive roles depending on the cancer types, yet there is no report regarding KLB's function in NSCLC. It is therefore intriguing for us to define the role of KLB in NSCLC.

In this study, we examined the expression levels of KLB in tumor vs. non-tumor tissues of NSCLC, explored its clinical relevance as a diagnostic/prognostic biomarker, and tested its antitumor effect both *in vitro* and *in vivo*. We propose KLB could serve as a novel target for theranostics in NSCLC.

## Materials and Methods

### Human lung cancer samples and cell lines

Lung cancer and their matched non-tumor tissue samples were obtained from Shanghai Jiao Tong University-affiliated Shanghai Chest Hospital, (Shanghai, China) after surgical resection. 84 serum samples including 57 from lung cancer patients and 27 from normal subjects were obtained from Shanghai Chest Hospital between January 2017 and November 2017. All the clinic pathological characteristics of theses samples were summarized in Supplementary Table 1. Written, informed consents approving the use of tissue samples for research purposes were obtained from lung cancer patients and normal subjects. The Institute Research Ethics Committee of School of Biomedical Engineering, Shanghai Jiao Tong University approved the study. Beas-2b, HFL-1, HCC15, HCC95, H520, H1703, SK-MES-1, H1975, H1581, A549 and H1299 cell lines were obtained from the American Type Culture Collection (ATCC).

### Molecular reagents and antibodies

Exogenous βKlotho were purchased from R&D systems (cat. no. 5889-KB). Ethanol, methanol, RNase A and propidium iodide (PI) were purchased from Sigma‑Aldrich. DAPI were purchased from Life Technologies (Mulgrave, VIC, Australia). KLB antibodies (1:1,000 for western blot analysis; 1:100 for IHC; cat. no. AF5889) were purchased from R&D systems. Tubulin and Ki-67 were purchased from Abcam Corporation. Other antibodies for western blot were obtained from Cell Signaling Technology.

### Quantitative real-time PCR (qRT-PCR) and western blot analysis

Cells were lysed for RNA or protein extraction and then subjected to qRT-PCR or western blot as previously described 25. The detailed sequence of each primer used in the whole study for qRT-PCR was summarized in Supplementary Table 2.

### Immunohistochemistry (IHC) and Immunofluorescence microscopy (IF)

For IHC, paraformaldehyde-fixed tissues were washed with PBS for three times. After blocking with 10% donkey serum for 1 h at room temperature, tissues were incubated with primary antibodies at 4 °C overnight. After three times washing, tissues were incubated with secondary antibodies (Abcam, ab6884) for 1 h at room temperature. Diaminobenzidine-hydrogen peroxide (Sigma) was the chromogen, and the counterstaining was carried out with 0.5% hematoxylin. The intensities of IHC staining was classified into the following grades:(-, no staining; +, 10%; ++, 10%-50%; +++, >50%). For IF, cells were seeded on coverslips in a 24-well plate and immunofluorescence staining was conducted as previously described 25. The primary antibody was goat anti-KLB. The secondary antibody was donkey anti-goat IgG conjugated with Alexa Fluor 594 (ThermoFisher, A-11058). Cell nuclei were stained with DAPI (Sigma) for 5 min at room temperature. Stained cells were photographed and quantified under an immunofluorescence microscope (Leica DFC420C). Two experienced pathologists scored the KLB staining independently, and the final score was the score average from both pathologists.

### Analysis of public datasets from Oncomine and the use of Kaplan-Meier Plotter

Relative copy number and mRNA levels of KLB of TCGA provisional LSQ and LADC cohorts and other lung cancer datasets were downloaded from Oncomine database (https://www.oncomine.org). Linear regression correlations between mRNA levels of KLB and Vimentin were conducted. Prognostic values of KLB mRNA levels were analyzed by Kaplan-Meier survival curves of NSCLC patients, using Kaplan-Meier Plotter (www.kmplot.com) 26. Log-rank test was used for statistical analysis.

### Gene Set Enrichment Analysis (GSEA)

Gene Set Enrichment Analysis was performed using Cbioportal database (http://www.cbioportal.org/datasets), in which two datasets were used, Lung Adenocarcinoma (TCGA, PanCancer Atlas) and Lung Squamous Cell Carcinoma (TCGA, PanCancer Atlas). Genes were ranked by the expression of KLB, then top 25% and bottom 25% of the genes were chosen to apply analysis in GSEA using KEGG and GO databases following the official user guide of GSEA 27.

### Lentivirus transduction and generation of stable cell lines

Human βKlotho overexpression lentivirus (KLB-OE) and negative control lentivirus (KLB-EV) were purchased from GENECHEM (Shanghai, China). Lentivirus transductions and stable cell line generation were performed as previously described 25.

### Cell Proliferation Assay

Cell proliferation was assessed by using Cell Titer 96® Aqueous One Solution Cell Proliferation Assay kit (Promega, USA). Briefly, cells were seeded in a 96-well plate at a density of 1,000 cells per well after transfection or in the presence or absence of KLB with different concentrations. After 72 h, medium was removed followed by washing with PBS twice. Then 10 µL Cell Titer 96® Aqueous One Solution and another 90 µL fresh medium per well was added to the 96-well plate. After 1-4 h incubation at 37 °C, the optical density was measured at 490 nm with a microplate reader (Synergy2, BioTek, Winooski, VT).

### Flow cytometry-based Annexin V/7-AAD assay

The flow cytometry assay was performed to measure the levels of apoptosis by using ApoScreen Annexin V kit (Southern Biotech, Birmingham, AL, USA) according to the manufacturer's protocol. Briefly, medium was collected followed by washing with cold PBS twice and the cold PBS was collected as well. Then cells were digested with 0.25% trypsin-EDTA to resuspend cells to a concentration between 1 × 10^6^ and 1 × 10^7^ cells/mL in cold 1X binding buffer. Later 5 µL fluorescence-labeled Annexin V was added into 100 µL suspended cells. After incubation on ice for 15 min in the dark, 200 µL binding buffer and 5 µL 7-AAD solution were added into the cell suspensions. The number of stained cells was assessed immediately by a flow cytometer (FACS Aria II, BD Biosciences).

### Cell cycle analysis

Before experiment, cells in the 6-well plate were grown to a density of 90%. Cancer cells were collected by trypsinization and assessed for cell cycle by flowcytometry as described with a modification. In brief, the cells were fixed with 70% cold ethanol at 4 °C overnight, then washed with PBS and treated with 100 μg/mL RNase A for 30 min at 37 °C, and stained with 50 μg/mL propidium iodide (PI) in the dark. Subsequently, the cells were analyzed by flow cytometry (BD FACS Calibur). At least 10,000 cells in each sample were analyzed to obtain a measurable signal. The cell debris and fixation artifacts were gated out and the populations of cells which were at the G0/G1, S and G2/M phases were quantified using the Modfit LT 4.0 software (BD Biosciences).

### Transwell migration/invasion assay

Cells were serum starved for 24 h, then harvested and re-suspended in serum-free medium and were seeded in an 8 μm pore membrane with or without Matrigel-coating (CORNING,356231), and with or without βKlotho (40 ng/mL). Serum-containing medium (600 µL) was added to the bottom of the well and the inserts were incubated for 24 h to 48 h. After incubation, cells were removed and the inserts were fixed in methanol for 20 min and then stained for 15 min with 0.1% crystal violet dye. The stained cells were counted at three randomly selected views for subsequent calculations.

### Colony formation

Cells were transfected with either KLB-OE lentivirus or control lentivirus, or by exogenous βKlotho administration (40 ng/mL), and cultured in media containing G418 for two weeks and stained to determine the number of surviving colonies.

### siRNA transfection

Cells were transfected with siRNAs using Lipofectamine RNAiMAX Reagent (Life Technologies) according to the manufacturer's protocol. The cells were incubated with the transfection complex, and the gene knockdown efficiency was assessed after 48 h. siRNAs were synthesized by OriGene Technologies. The KLB siRNA-1 sequence was 5'GCAAUAAGGUUAGAUGAAAUACGAG3', the KLB siRNA-2 sequence was 5'GGAUUAAACUGGAAUACAACAACCC3', and the control siRNA sequence was 5'UUCUCC GAACGUGUCACGUTT3'.

### Lung cancer xenograft model

BCLB/C nude mice were subcutaneously injected into the right flanks with a total of 5 × 10^6^ HCC15 cells in a volume of 50 µL. Tumor sizes and body weights were measured every 3 days. All the mice were raised in the specific pathogen free animal room of Shanghai Jiao Tong University. At day 28, tumors were harvested, cut into 3 mm pieces, and implanted into mice subcutaneously again (n = 14). All the tumor pieces were of the same size originally and mice were randomly grouped for further study, in which KLB-OE lentivirus (1 × 10^7^ in 2 µL) and the control lentivirus was injected intratumorally once a week for four weeks before harvesting the tumors. After the injection, the needle was left in place for 5 min to prevent regurgitation of the virus during removal.

### *In vivo* orthotopic lung cancer model

Orthotopic lung cancer model was modified from our previous study 28. In brief, a 5 mm incision was sheared on the dorsal side over left lung, 0.5 cm below the scapula on the 4-week-old male BCLB/C nude mice. Cell suspension of H1581 (1 × 10^6^ cells) in a total volume of 50 µL (PBS: Matrigel = 4:1) were injected directly into the left lateral lung with insulin injection syringes.

### Enzyme-linked immunosorbent assay (ELISA)

Blood samples were processed within one hour after collection and stored at -80 °C until analysis. Serum concentrations of βKlotho were evaluated using ELISA kits (R & D, DY5889-05), following the manufacturer's instructions.

### Statistical analysis

All statistical analyses were performed using the GraphPad Prism 5 software. Data were presented as mean ± SD, and the paired or unpaired Student's t-test or ANOVA were chosen to analyze the statistical significance between two groups. P-values < 0.05 was considered statistically significant.

## Results

### Downregulation of KLB levels in tumor tissues of NSCLC

**Figure 1:**
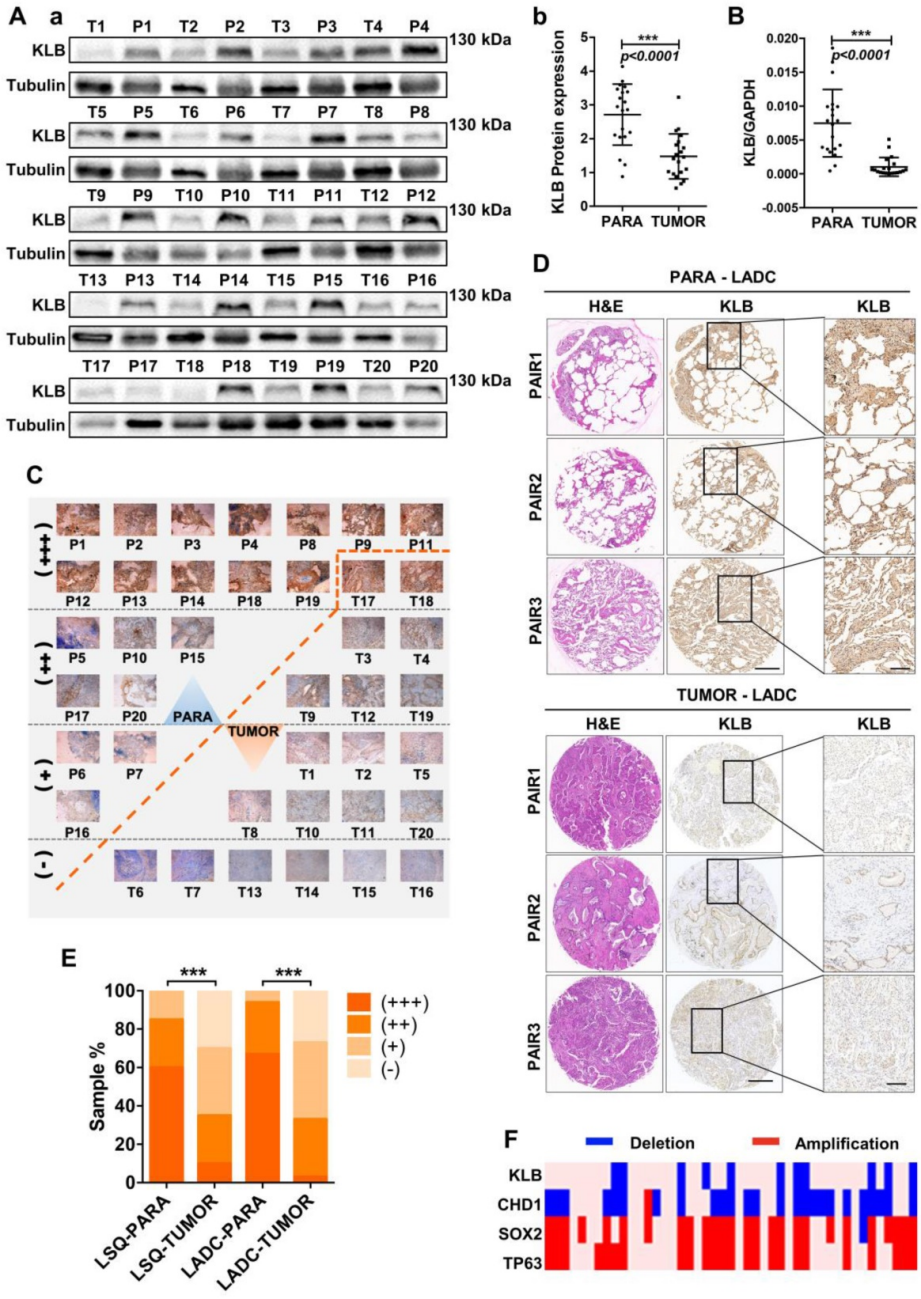
** KLB expression is reduced in human NSCLC compared to adjacent non-tumor tissues. A. (a)** Protein levels of KLB in 20 LSQ samples (T) and its paired Para-tumor tissues (P) by Western blot analysis. **(b)** Densitometric analysis KLB protein levels (normalized to tubulin). **B.** KLB mRNA levels were determined by qRT-PCR in LSQ samples relative to its matched non-tumor tissue (normalized to GPADH). **C.** IHC staining of KLB in all the 20 LSQ and paired non-tumor samples. **D.** Representative images of immunohistochemistry of KLB in tumor and para-tumor tissues from LADC samples (n = 30 per group). Scale bars, 500 µm, and enlarged scale bars, 100 µm. **E.** Quantification of IHC staining intensity for KLB in paired lung squamous cell carcinoma (LSQ) (n = 20) and lung adenocarcinoma (LADC) (n = 30). +++, extremely positive; ++, strongly positive; +, positive; -, negative. **F.** Heat map of copy number gains and deletions in 37 LSCC patients. Each column denoted an individual normal/tumor paired patient, and each row represented a gene (student's t-test, * **P < 0.001).

To explore the relationship between KLB expression levels and NSCLC progression, we examined the KLB expression in 20 lung squamous cell cancer (LSQ) samples and 30 lung adenocarcinoma (LADC) samples along with matched non-tumor control samples. Western blot analysis showed reduced KLB expression in LSQ when compared to control samples (Figure 1A), and this was verified by qRT-PCR (Figure 1B). All of these 20 LSQ samples were also analyzed by IHC staining with an antibody against KLB, and based on the intensity of the staining, samples were categorized into extremely positive (+++), strongly positive (++); positive (+) and non-detectable (-) categories. Expectedly, overall tumors exhibited decreased KLB staining compared to non-tumor samples (Figure 1C, E). More specifically, 60% (12/20) of all the non-tumor samples were found to express high levels of KLB, whereas KLB was barely detectable in 30% (6/20) of all the LSQ tissues.

IHC was performed to detect KLB protein levels in another 30 sets of LADC samples. Consistently, decreased KLB levels were detected in LADC tissues compared with the paired neighboring noncancerous tissues, and representative staining of 3 pairs of tumor/non-tumor tissues was shown (Figure 1D). Similar to LSQ samples, LADC showed lower levels of KLB vs. non-tumor tissues (Figure 1D, E). For instance, in the 21 sections that stained KLB as extremely positive, 20 (20/30) were from the non-tumor tissue group and only 1 (1/30) was from LADC tissue group (Figure 1E).

We also detected copy number variations of KLB in 37 LSQ samples that were sequenced for an earlier study by our group 29. Through the comparative analysis between tumor and matched adjacent normal tissue, we identified large-scale amplification of SOX2 (26/37) and TP63 (24/37) and deletion of CDH1 (25/37) in tumor tissues. It was noted that KLB exhibited a deletion rate of 29.7% (11/37) in our cohort, indicating a relatively high frequency of DNA level changes (Figure 1F). Collectively, these results from clinical samples indicated that expression of KLB was downregulated in NSCLC.

### Characterization of KLB expression, copy number variation and its relevance with NSCLC progression in clinical datasets

**Figure 2:**
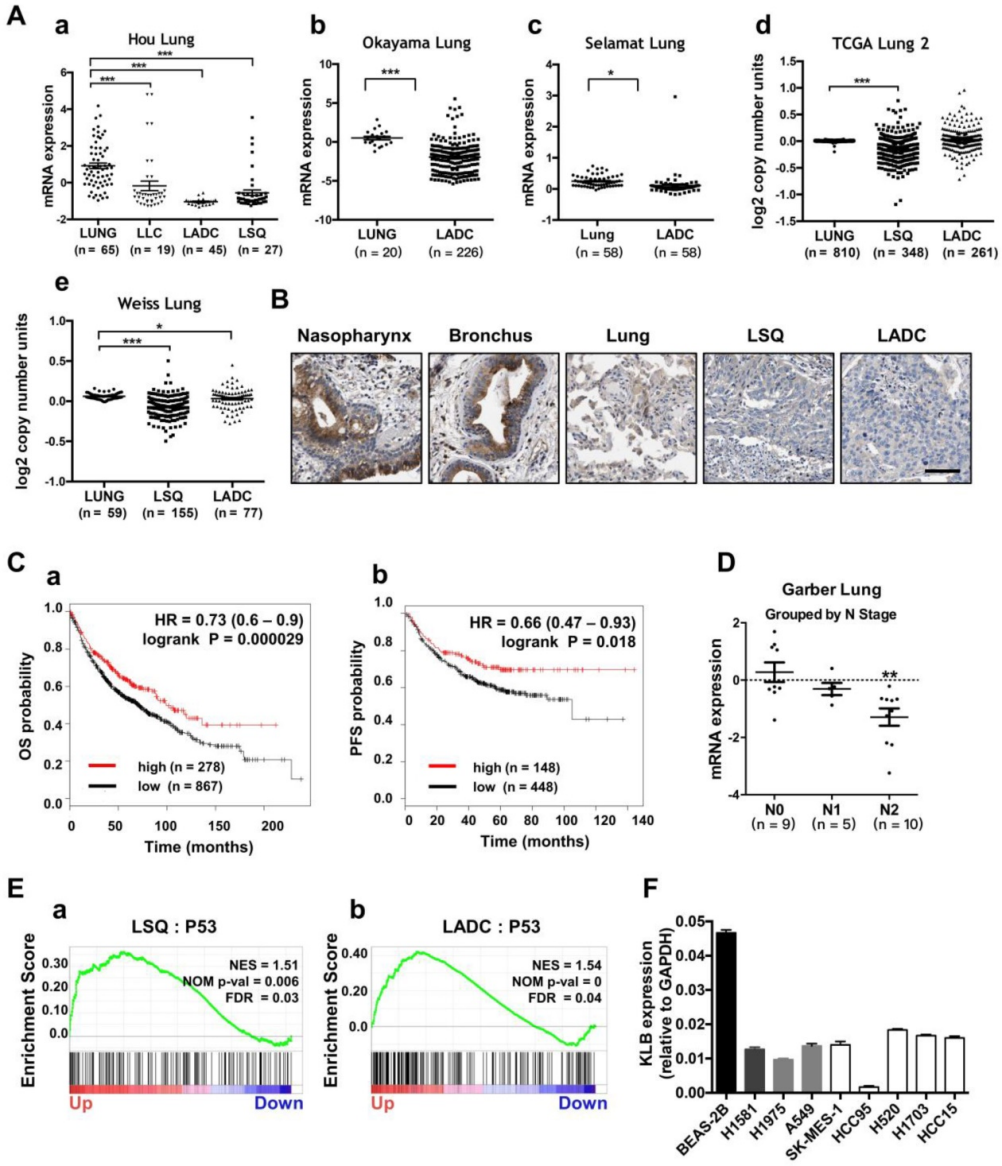
** Lower expression of KLB was associated with shorter OS and PFS in NSCLC and KLB was down-regulated in human metastatic lung cancer. A.** Expression and copy number analyses of KLB in Oncomine database with provisional LSQ, LADC and LLC cohorts. Expression (a) in Hou Lung datasets, (b) in Okayama Lung datasets, (c) in Selamat Lung datasets, copy number (d) in TCGA Lung2 datasets, (e) in Weiss Lung datasets. **B.** IHC of KLB in different parts of lung associated tissues (https://www.proteinatlas.org), Scale bar = 50 μm** C.** Kaplan-Meier analysis of KLB mRNA expression level (higher KLB level: top 25%, lower KLB level: bottom 25%) on the (a)overall survival (OS; n = 1145) and (b) progression free survival (PFS; n = 596) in lung cancer patients (http://www.kmplot.com). **D.** Expression of KLB grouped by N-stage of samples in Garber Lung datasets from Oncomine database with provisional LSQ and LADC cohorts. **E.** Enrichment plots of P53 expression signatures according to KLB expression levels in a lung cancer cohort. **F.** KLB mRNA expression in NSCLC cell lines and bronchial epithelial cell line Beas-2b. (Student's t-test, * P < 0.05, ***P < 0.001).

To evaluate the prognostic value of KLB in lung cancer patients, we first analyzed the expression and copy number features of KLB in the TCGA provisional LSQ and LADC cohort in Oncomine database. Decreased KLB expression levels were detected in Hou Lung datasets (Figure 2A a), Okayama Lung datasets (Figure 2A b) and in Selamat Lung datasets (Figure 2A c) both in LSQ and LADC cohorts. Consistently, KLB copy number was also decreased compared to the normal lung tissues in TCGA Lung2 datasets (Figure 2A d) and in Weiss Lung datasets (Figure 2A e). All these results were in accordance with analyses on our own clinical samples (Figure 1). To further verify the level of KLB in the development of lung cancer, we searched IHC data of KLB in different parts of lung-associated tissues (https://www.proteinatlas.org). High levels of KLB in respiratory epithelial cells of nasopharynx and bronchus were detected, whereas in macrophages of lung tissue, KLB showed mild expression and LSQ and LADC samples exhibited extremely low expression (Figure 2B).

We then analyzed the Kaplan-Meier plots and found that higher KLB level (top 25%) was associated with longer overall survival (OS; n = 1145) and progression-free survival (PFS; n = 596) vs. lower KLB level (bottom 25%) in NSCLC (Figure. 2C). In LSQ and LADC metastasis cohort, we also found KLB downregulation of expression in Garber Lung datasets respectively by N stage (Figure 2D). Moreover, gene set enrichment analysis (GSEA) plots for KLB in LSQ and LADC revealed that P53 was enriched in KLB up genes (Figure 2E), which might reflect that upregulation of KLB may have effect on cancer. Furthermore, KLB down genes were identified, which involved those in cell cycle, DNA replication and packaging, cell division, WNT signaling and other cancer-related pathways such as MYC, VEGF-A and MTOR (Figure S1). We then evaluated mRNA levels of KLB in NSCLC cell lines and a normal bronchial epithelial cell line Beas-2b. In agreement with our clinical findings, KLB was downregulated in these NSCLC cell lines (Figure 2F).

### KLB inhibited the proliferation of NSCLC cells

**Figure 3:**
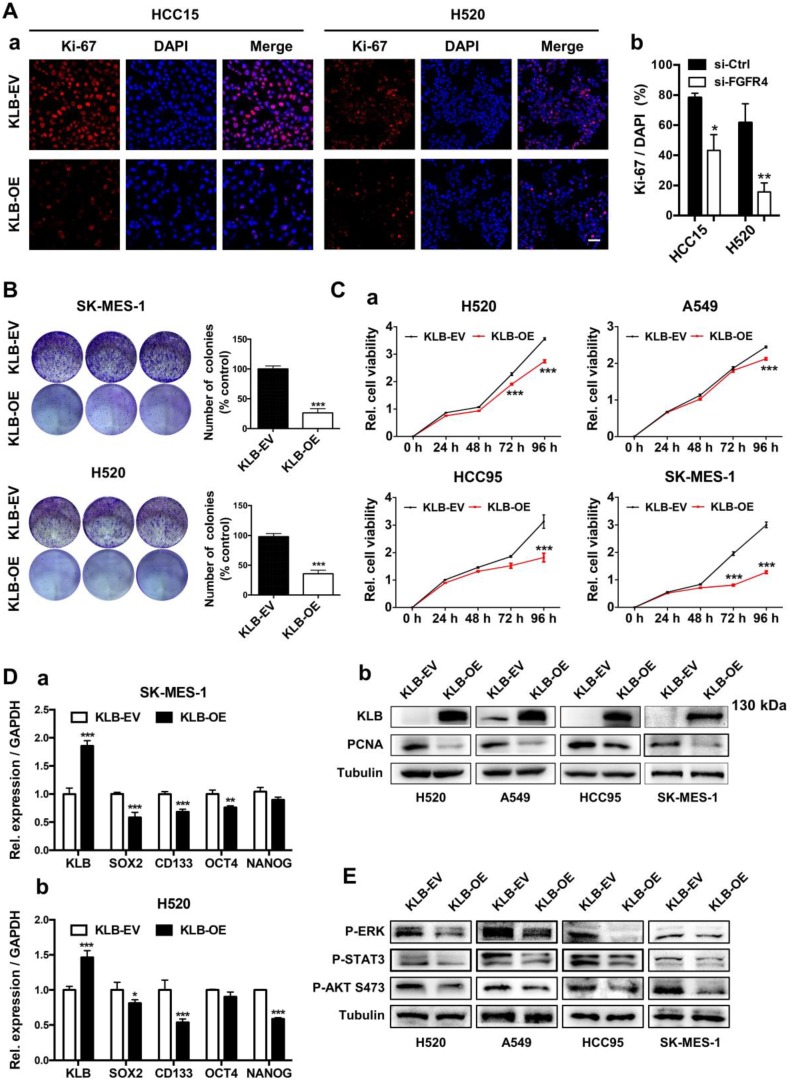
** High levels of KLB inhibit the proliferation and inhibits ERK, STAT3, AKT pathway of human NSCLC cells. A.** Cancer cells were transfected with a pCDH-KLB expression vector (KLB-OE) or a control pCDH vector (KLB-EV). IF staining of Ki67(red) and DAPI (for nucleus, blue) in HCC15 and H520 cells after transfection. Scale bar = 50 μm. The right panel was % of Ki-67 positive cells. **B.** Colony formation in SK-MES-1 and H520 cells. **C. (a)** Cell viability assay of stably overexpressed (KLB-OE) and control (KLB-EV) cancer cells. **(b)** Protein levels of KLB and proliferation marker PCNA were determined by western blot. **D.** qRT-PCR analysis of stemness-related genes in **(a)** SK-MES-1 cells and **(b)** H520 cells. E. Overexpression of KLB suppressed ERK, STAT3, AKT pathway. Data were represented as mean and SEM from three independent experiments. * P < 0.05. ** P < 0.01. *** P < 0.001.

Since there was an inverse correlation between the expression of KLB and NSCLC progression, we further explored the functional role of KLB in NSCLC. We first constructed the KLB overexpressing vector (KLB-OE) which was also packaged into lentiviral system. After transient transfection of KLB-OE into HCC15 and H520 cells, the effect of KLB overexpression in cell proliferation was measured by immunofluorescence staining of Ki-67 (Figure 3A). The percentage of Ki-67 positive cells was reduced in the KLB-OE group by more than 65% vs. the KLB empty vector (KLB-EV) control group. Next, a panel of NSCLC cell lines including H520, A549, HCC95 and SK-MES-1 were stably transduced with KLB-OE lentivirus vectors. In SK-MES-1 and H520 cells, KLB overexpression led to a significant reduction of colony formation by more than 50% (Figure 3B) and in all these four cell lines, significant inhibition of cell growth was found (Figure 3C), along with a significantly reduction of PCNA level. Furthermore, qPCR results showed that overexpression of KLB decreased the mRNA levels of stemness-related markers SOX2, CD133, OCT4 and NANOG in SK-MES-1 and H520 cell lines (Figure 3D), corroborating the colony formation experiments. Overexpression of KLB also inhibited ERK, STAT3, AKT pathway (Figure 3E), which are important signals for cell survival and proliferation. Taken together, these results indicated that overexpression of KLB inhibited the proliferation of NSCLC cells.

### KLB overexpression induced apoptosis and G1 to S phase arrest of NSCLC cells

**Figure 4:**
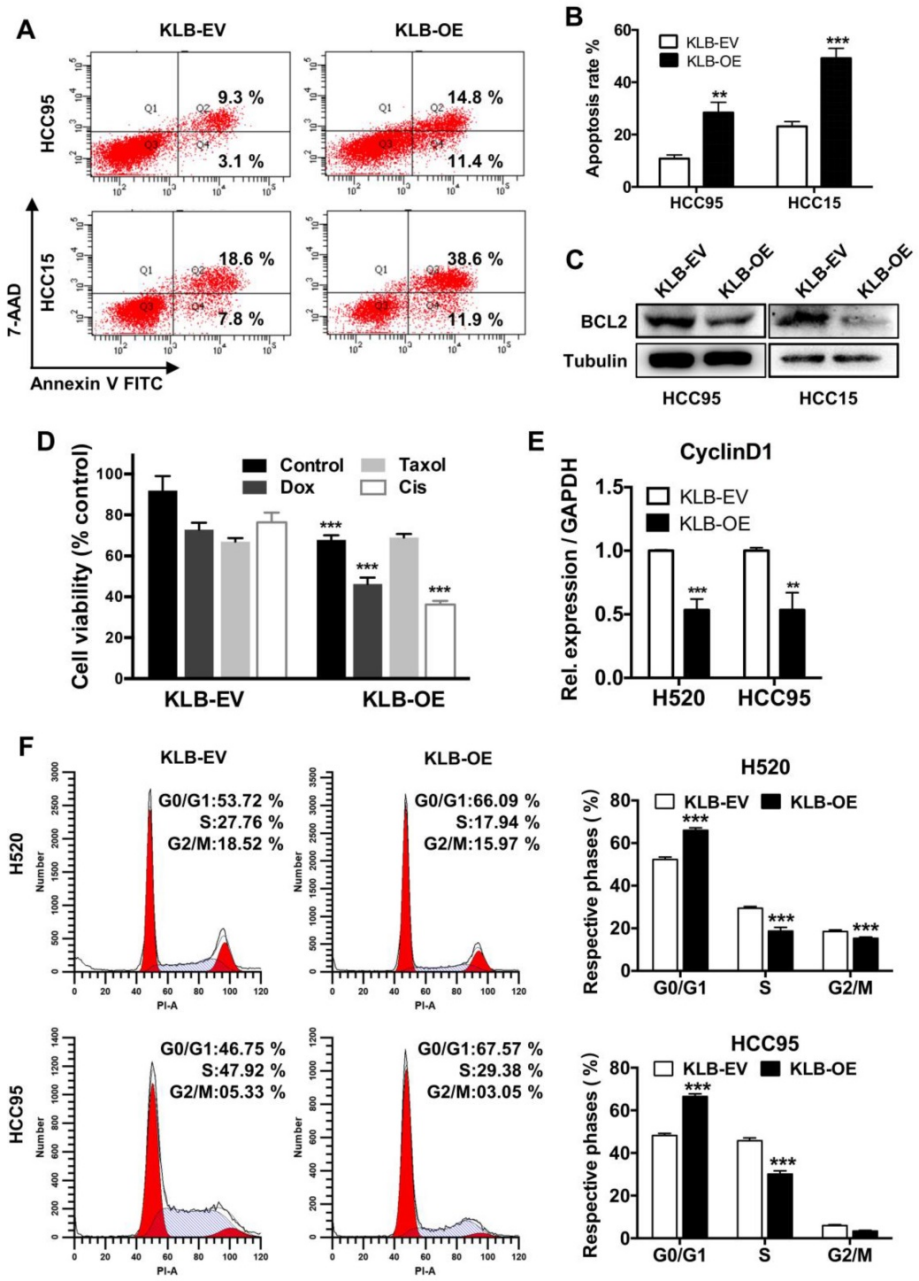
** KLB overexpression promoted apoptosis and arrested cell cycle. A.** KLB overexpression induced apoptosis. **B.** Quantifications of the results from the FACS-based study. **C.** Protein levels of anti-apoptosis marker BCL-2 were determined by western blot. **D.** The HCC15 cells were incubated with the indicated concentrations of control (DMSO), Dox (5 μM), Taxol (250 nM), Cisplatin (500 nM) for 48 h after transfection with KLB-OE or KLB-EV. Cell viability was determined by CCK-8. **E.** qRT-PCR analysis of cell-cycle related gene CyclinD1 in H520 and HCC95 cells. **F.** Representative histograms depicting cell cycle profiles of HCC95 cells and H520 cells transfected with KLB-OE or KLB-EV. Right panel: Quantifications of the histograms. Data were collected from three independent experiments. *p < 0.05. ** P < 0.01. ***P < 0.001.

We further conducted FACS-based Annexin V/7-AAD assay to determine whether KLB induced apoptosis in NSCLC cells. Overexpression of KLB in HCC95 and HCC15 cells significantly increased the percentage of the cells in both early-stage and late-stage apoptosis (Figure 4A and B). We then analyzed the levels of BCL-2, which is also one of the most important oncogenes involved in cancer for inhibiting apoptosis 30. BCL-2 levels in both KLB overexpressed cell lines were decreased (Figure 4C). Since KLB overexpression induced cell apoptosis, we hypothesized that KLB could enhance the sensitivity of NSCLC cells to anti-cancer drugs. Doxorubicin, Taxol and Cisplatin are commonly used chemotherapeutics. HCC15 cells, transfected with either KLB-EV or KLB-OE, were treated with Doxorubicin (5 μM), Taxol (250 nM) and Cisplatin (500 nM) or a vehicle control (DMSO) for 48 hours, followed by CCK-8 viability assay. Overexpression of KLB significantly increased the anti-tumor effects of Doxorubicin and Cisplatin (Figure 4D). As we previously observed that PCNA decreased along with KLB up-regulation (Figure 3Cb), we then investigated whether KLB could influence cell cycle. We first detected the expression of cyclin D1, a critical regulator of the transition from G1 to S phase in cell cycle. Cyclin D1 expression was dramatically down-regulated in KLB-overexpressed cells (Figure 4E). Moreover, overexpression of KLB in H520 and HCC95 cells increased the percentage of cells in G0/G1 but decreased the percentage of cells in S stage (Figure 4F), indicating that KLB overexpression induced G1 to S phase arrest of NSCLC cells. To summarize, these data suggested the anti-proliferative effect of KLB was associated with induction of apoptosis and G1 to S phase arrest.

### Overexpression of KLB inhibited migration, invasion and EMT of NSCLC cells

**Figure 5:**
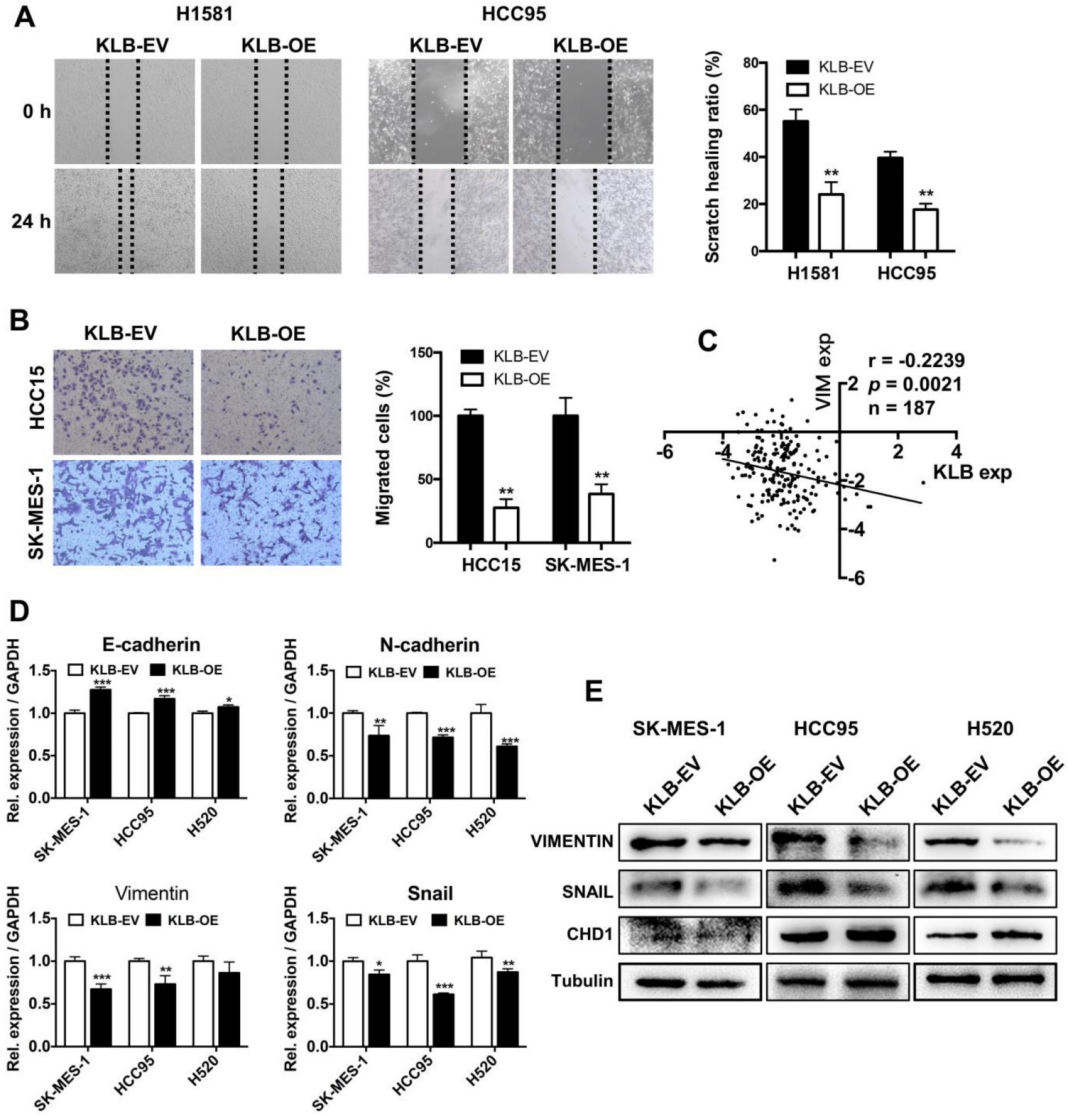
** Overexpression of KLB inhibited migration and invasion and EMT of NSCLC cells.** Cells were treated with KLB-OE or KLB-EV accordingly, after 24 h **(A)** scratch and **(B)** transwell assays were conducted to access cell migration and invasion. **C.** Vimentin and KLB showed negative relationship in Oncomine TCGA datasets. **D-E.** SK-MES-1, HCC95 and H520 cells were pretreated with KLB-OE and KLB-EV accordingly, after 72 h, expression of EMT markers E- cadherin, N-cadherin, snail and Vimentin were analyzed by qPCR and western blot. Data were representative of three independent experiments. P values were calculated using paired t-test. *p < 0.05; **p < 0.01; ***p < 0.001.

To investigate whether KLB overexpression could affect migration, invasion and EMT in NSCLC cells, we conducted following experiments. H1581, HCC95, HCC15 and SK-MES-1 cells were scratched or re-seeded into the transwell plates after transfection with KLB-OE or KLB-EV respectively for 24 h. Compared with the control group, the migration (Figure 5A) and invasion (Figure 5B) were significantly reduced in the KLB-OE group. From Oncomine TCGA data, we found a negative relationship between VIMENTIN and KLB (Figure 5C). Moreover, SK-MES-1, HCC95 and H520 cells that were treated with KLB-OE vector expressed less VIMENTIN and SNAIL and more E-cadherin in both mRNA level (Figure 5D) and protein level (Figure 5E). Altogether, these results indicated that upregulation of KLB could inhibit the migration, invasion and EMT in NSCLC cells.

### Downregulation of KLB facilitated a tumor-promoting phenotype in NSCLC cells

**Figure 6:**
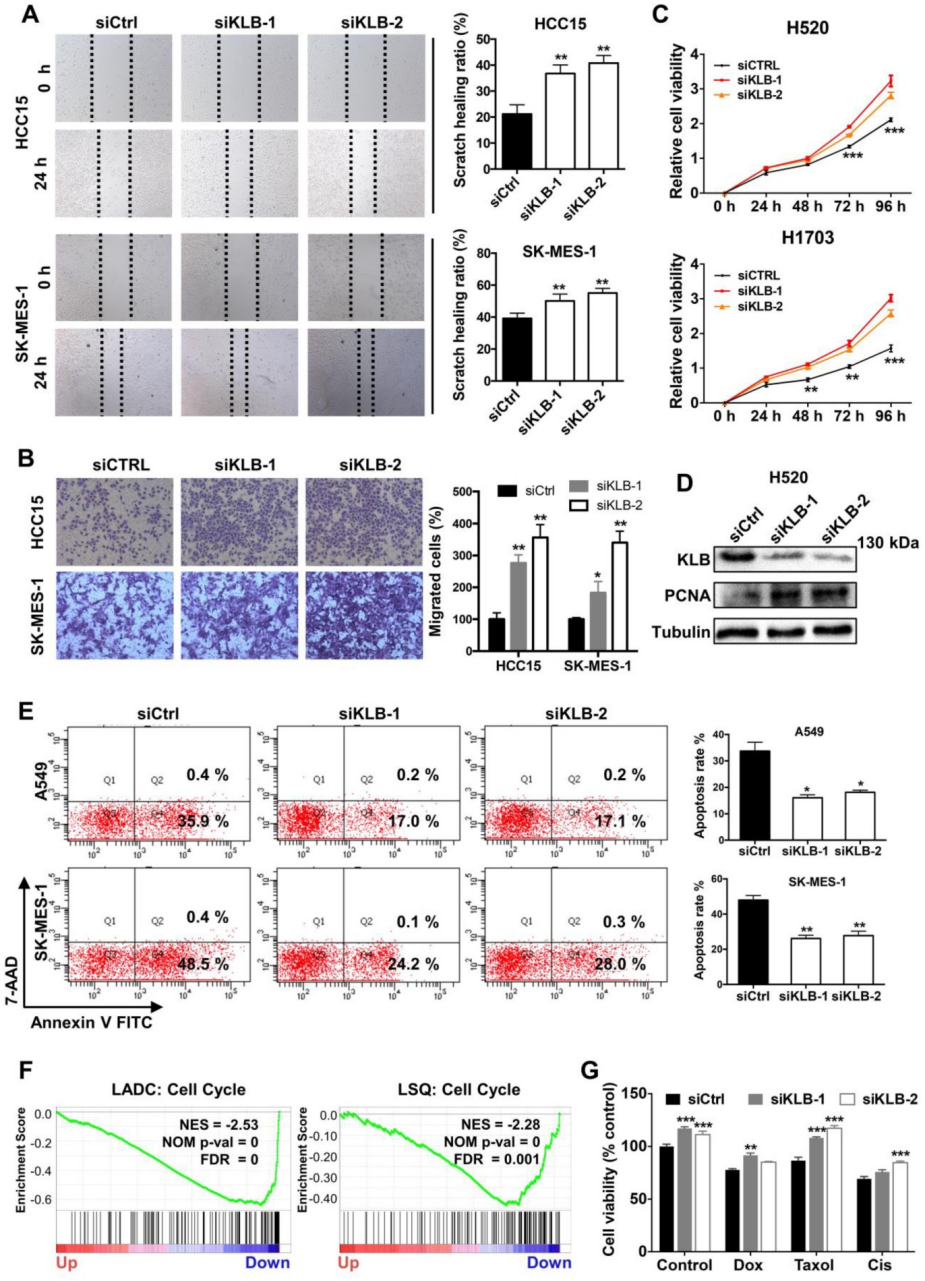
** Downregulation of KLB facilitated a tumor-promoting phenotype in NSCLC cells.** Cells were transfected with either control siRNA or KLB siRNA. Scratch **(A)**, transwell** (B)**, and CCK-8 **(C)** assays were conducted to access cell migration, proliferation and invasion. **D.** Protein levels of KLB and proliferation marker PCNA were determined by western blot. **E.** In A549 and SK-MES-1 cells, after transfection for 72 h, medium was replaced by 1 μM H_2_O_2_ for 30 min, FACS- based Annexin V/7-AAD assays were then conducted to determine apoptosis (left panel). Quantifications of the results from the FACS-based study (right panel). **F.** Enrichment plots of cell cycle according to KLB expression levels in a lung cancer cohort. **G.** The HCC15 cells were incubated with the indicated concentrations of control (DMSO), Dox (5 μM), Taxol (250 nM), Cisplatin (500 nM) for 48 h after transfection. Cell viability was determined by CCK-8. Data were collected from three independent experiments. Scale bars = 100 µm. *p < 0.05. ** P < 0.01. ***P < 0.001.

To further demonstrate the function of KLB in the progression of NSCLC, we transfected cells with either control siRNA or KLB siRNA. We determined that downregulation of KLB increased migration (Figure 6A) and invasion (Figure 6B) of HCC15 and SK-MES-1 cells and stimulated cell proliferation in H520 and H1703 cells (Figure 6C). Downregulation of KLB in H520 exhibited a higher level of PCNA (Figure 6D). Furthermore, we found that knockdown of KLB in A549 and SK-MES-1 cells could attenuate hydrogen peroxide-induced apoptosis by over 50% than the control group (Figure 6E). We also found that KLB was negatively associated with cell cycle related genes in a lung cancer cohort (Figure 6F). Moreover, KLB-knockdown HCC15 cells became less sensitive to chemotherapeutic drugs including Doxorubicin, Taxol and Cisplatin (Figure 6G). Taken together, these findings have shown that a KLB-deficiency renders a tumor-promoting phenotype in NSCLC cells.

### KLB overexpression repressed the tumorigenicity and growth of NSCLC cells *in vivo*

**Figure 7:**
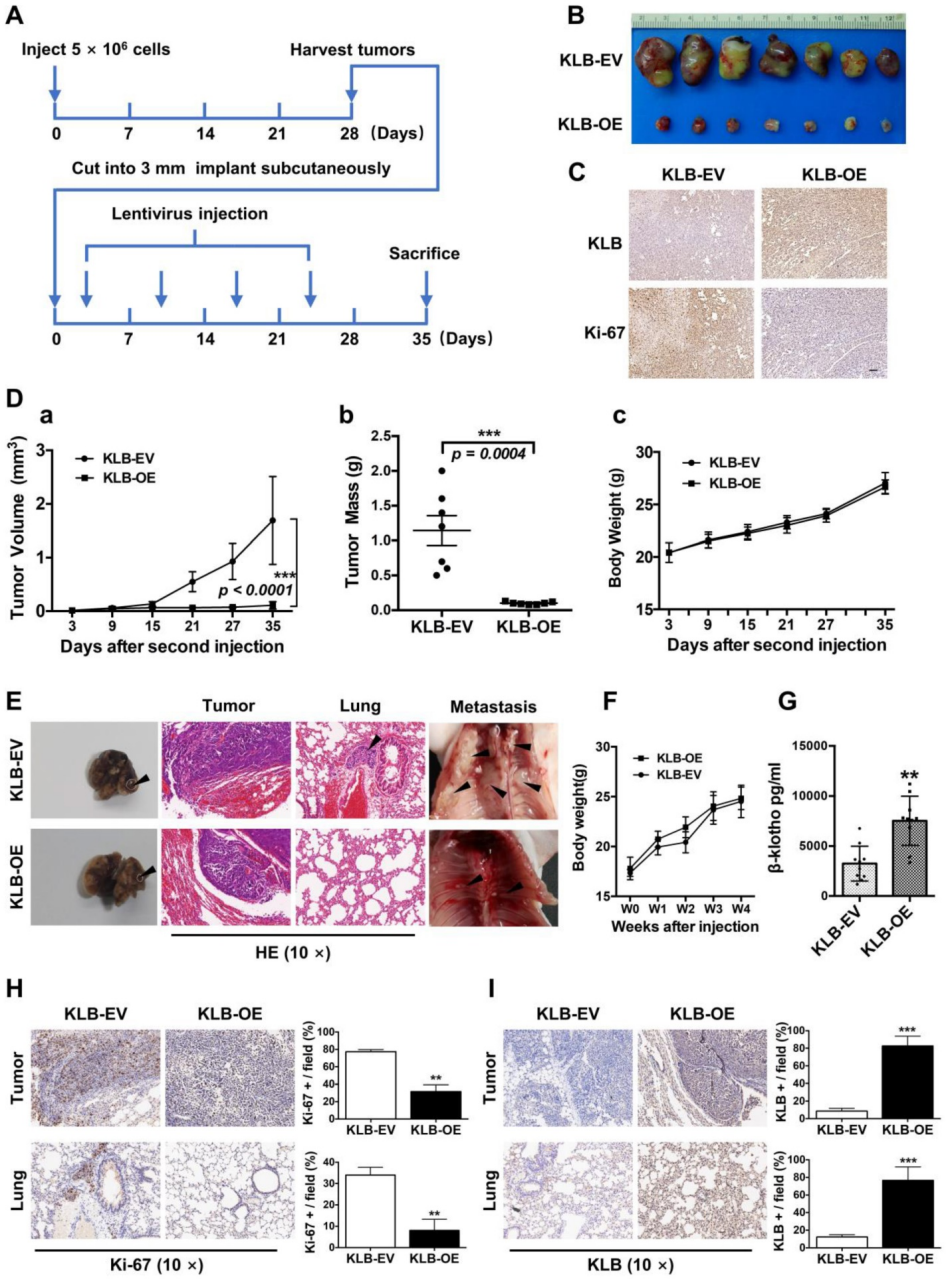
** KLB overexpression repressed the tumorigenicity and growth of NSCLC cells *in vivo*. A.** Schematic representation of mouse model.** B.** Images of tumor nodules from subcutaneous mouse xenograft model. **C.** IHC analysis of KLB and Ki-67 expression in tumors. **D. (a)** Volume of tumors, **(b)** weight of tumors and **(c)** body weight of mice from two groups. **E.** Representative images of primary (left panel), HE staining (middle panel) and metastatic (right panel) tumors in the orthotopic lung cancer models from each group after implantation of H1581 cells stably transfected with KLB-OE or KLB-EV lentivirus. **F.** Mice body weight from two groups. **G.** Serum levels of βKlotho from two groups. **H.** IHC analysis of Ki-67 expression in tumors and lungs. **I.** IHC analysis of KLB expression in tumors and lungs. Scale bars = 100 µm. **P <0.01, ***P <0.001.

The *in vitro* data prompted us to investigate the role of KLB in NSCLC *in vivo*, Using a subcutaneous model (Figure 7A), a marked reduction in tumor growth was observed in mice receiving KLB-OE lentivirus compared with those receiving the control lentivirus (Figure 7B). This effect was also accompanied by lower Ki-67 expression (Figure 7C), smaller tumor volume (Figure 7D a), and decreased tumor weight (Figure 7D b) in the treatment group vs. control group, but with no difference in body weight between the two groups.

To test the role of KLB in a physiologically more relevant tumor background, we developed orthotopic mouse models for lung cancer that closely recapitulate the clinical features of human lung cancer by using H1581 cells that was stably infected with KLB-OE lentivirus or control lentivirus (n = 10 per group). At the fourth week, we sacrificed mice from each group and analyzed the tumors. Compared with KLB-EV group, overexpression of KLB significantly suppressed the progression of tumors (Figure 7E, left panel) and we detected more metastatic nodules in the KLB-EV group (Figure 7E, middle and right panel). Mice body weight between the two groups showed no significant differences (Figure 7F). Serum levels of βKlotho in KLB-OE group were significantly higher than those in control subjects (Figure 7G). We further performed IHC staining for Ki-67 (Figure 7H) and KLB (Figure 7I) of both the tumor and the lung tissues, and found a remarkable loss of proliferation in tumors of the KLB-OE groups. Taken together, these observations demonstrated that KLB could suppress tumor growth *in vivo*.

### Serum βKlotho exhibited as a diagnostic marker and exogenous βKlotho suppressed tumor phenotype in NSCLC cells

**Figure 8:**
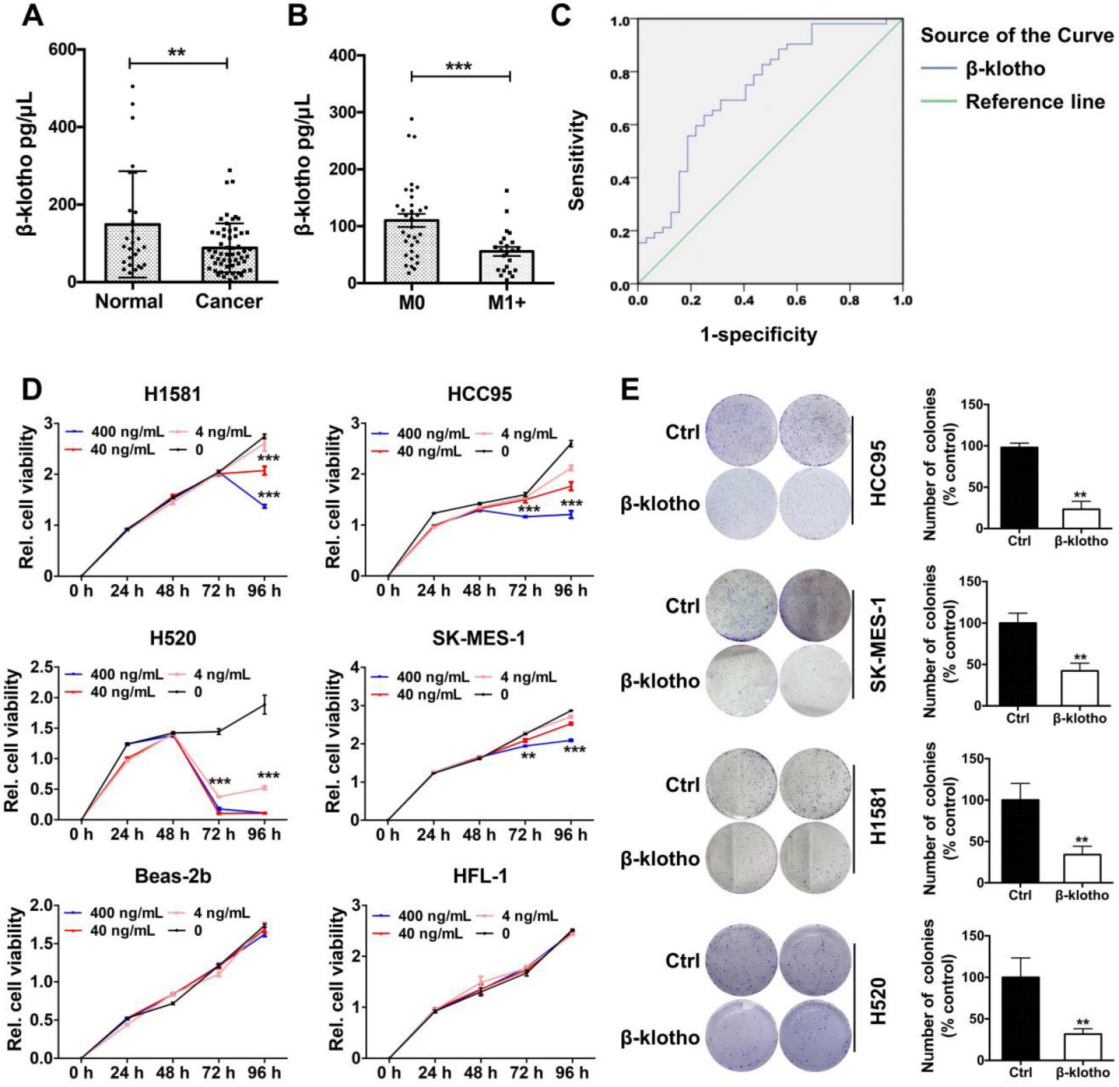
** Serum βKlotho exhibited as a diagnostic marker and exogenous βKlotho suppressed tumor phenotype in NSCLC cells. A.** Serum levels of βKlotho in NSCLC patients (n = 57) were significantly lower than in control subjects (n = 27). **B.** Within 57 NSCLC patients, serum levels of βKlotho in patients with metastasis (n = 34) were lower than patients without metastasis (n = 23). **C.** Receiver operating characteristic (ROC) curve analysis of serum βKlotho levels in 57 NSCLC patients vs. 27 controls (*P<0.05). **D.** βKlotho was added to cultures cells in serum free-medium at the indicated concentration and cell number analyzed at either 72 or 96 h depending on the cell line by CCK-8. **E.** Colony formation assay were tested at a concentration of 400 ng/mL in different NSCLC cells. Right panel: quantifications of the colony formation results.

As βKlotho also exists in secreted, free flowing form 17, we further analyzed serum levels of βKlotho in NSCLC patients and control subjects. Serum levels of βKlotho in NSCLC patients were significantly lower than in control subjects (Figure 8A). Moreover, within the 57 NSCLC patients, serum levels of βKlotho in patients with metastasis (n = 34) were lower than patients without metastasis (n = 23) (Figure 8B). Also, receiver operating characteristic (ROC) curve analysis showed that when serum level of KLB was lower than 89.9 ng/mL, the sensitivity predicting was 63.5% and the specificity was 75% (Figure 8C). Moreover, when combined with other biomarkers in serum, the sensitivity could further increase to 88.5% (data not shown). Thus, serum βKlotho level might be a useful indicator for NSCLC.

We then tested whether exogenous βKlotho could inhibit the proliferation and colony formation of NSCLC cells. We added recombinant βKlotho to H1581, 1CC95, H520, SK-MES-1, Beas-2b and HFL-1 cells cultured in serum-free medium at different concentrations (0, 4, 40, 400 ng/mL). Proliferation of all four NSCLC cell lines was suppressed after βKlotho addition at either 72 or 96 h depending on the cell line, while other two non-tumor cell lines, Beas-2b (bronchial epithelial cells) and HFL-1 (lung fibroblast cells) were barely affected even at the concentration of 400 ng/mL (Figure 8D). Then we used these four lung cancer cell lines to test colony formation with the treatment of βKlotho at a concentration of 400 ng/mL. As expected, exogenous KLB decreased colony formation of NSCLC (Figure 8E).

In addition, we also tested whether exogenous βKlotho affected NSCLC apoptosis and cell cycle. We added exogenous βKlotho to cultured HCC95 and SK-MES-1 cells. After culturing for 72 h, FACS- based Annexin V/7-AAD assays were conducted to determine apoptosis (Supplementary Figure 2A), and a great increase of apoptotic signal was detected after βKlotho addition. Also, for H520 cells and HCC95 cells cultured with exogenous βKlotho for 72 h, βKlotho led to a significant increase in the percentage of cells in G0/G1 phase, as well as a significant decrease in the percentage of cells in S phase (Supplementary Figure 2B). In summary, exogenous βKlotho showed the similar tumor-suppressive effect to endogenous overexpression of KLB.

## Discussion

The purpose of our study was to investigate the role of KLB in NSCLC, and evaluate whether it could serve as a new target for theranostics. We showed for the first time that KLB was frequently downregulated in LSQ and LADC compared with adjacent non-tumor tissues and revealed an association between βKlotho serum levels and tumor metastasis. Decrease in KLB is positively correlated with N stage and have a shorter OS and PFS. Reintroduction of KLB into NSCLC cells inhibited their proliferation and promoted cell apoptosis. Furthermore, the anti-proliferation effect of KLB might be linked with G1 to S phase arrest, possibly through the downregulation of cyclin D1. KLB knockdown, however, even further promoted tumorigenic effect on NSCLC cells *in vitro*. Overexpression of KLB could suppress tumorigenesis in the subcutaneous and orthotopic mouse models, and exogenous βKlotho also showed the same effect as KLB overexpression in NSCLC cells. For successful treatment of NSCLC, drug resistance is considered as a major obstacle. Y. Wang et al. reported that klotho sensitized human lung cancer cell lines to cisplatin via PI3k/Akt pathway 31. Here in this study, we found that KLB treatment along with doxorubicin and cisplatin had a synergistic effect. We also found KLB copy number reduction and downregulation of expression by N stage in Oncomine database, and upregulation of KLB could inhibit the metastasis of cancer cells *in vivo*. These findings suggested KLB had an anti-tumorigenic role in NSCLC.

Our findings have several implications. First, serum levels of βKlotho were significantly downregulated in NSCLC. Thus, low expression of KLB may serve as a novel diagnostic index for screening, although a larger sample size is needed in future study. Second, the low expression of KLB in NSCLC played an important role in predicting the outcome in patients with NSCLC. Third, exogenous βKlotho or reintroduction of KLB inhibited NSCLC progression and it may achieve therapeutic potential for NSCLC. Hence, like Klotho 32, KLB is identified as a novel target for theranostics in NSCLC. Certainly, more study is needed to develop the KLB-based assays for diagnostics/prognostics analyses in NSCLC, and moreover, methods to reintroduce KLB to NSCLC should also be tested in larger scale animal studies. Nonetheless, the current study has proposed this new role of KLB as a target for theranostics in NSCLC.

Negative tumor markers are genes that code for proteins which inhibit cell proliferation and tumor development 33. In many tumors, they are lost or inactivated due to mutation(s), making proteins less effective at controlling cell growth and/or repair. In lung cancer, several tumor suppressor function as biomarkers for metastasis and prognosis, such as Rb, P53, PTEN 33, KAI1/CD82 34, PARK2 35 and Rad9 36. A number of studies have also identified aberrant microRNA expression in cancer. Expressions of miR-34,miR-200, miR-126, miR-195, miR218 37, and several other microRNAs and lncRNAs 38 were decreased in lung cancer, and these non-coding RNAs are very promising biomarkers in non-invasive screening methods. We identified that KLB is downregulated in NSCLC and KLB might be a negative tumor marker in NSCLC.

The mechanism of this tumor suppressive effect of KLB in NSCLC seems to be complicated. KLB has been identified as a FGFR4 co-receptor, which is required for high affinity binding and activity of FGF19 in specific liver functions 39. In our previous study, we found that in smoking LSQ patients, FGF19 was upregulated, consistent with the upregulation of its receptor FGFR4 (data not shown). The current study revealed the downregulation of co-receptor KLB in NSCLC. It appeared that the FGF19/FGFR4 signaling could still function in lung cancer independent of KLB. Indeed, it was demonstrated that FGF19, in high concentration, could still activate FGFR4 in a heparan-dependent manner even in the absence of the KLB 40. The mechanism underlying the expression regulation of the members in the FGF19/FGFR4/KLB axis should also be investigated. For instance, like methylation of the KL promoter region, how epigenetic regulation of KLB expression in relation to FGF19/FGFR4 signaling during lung cancer progression remains an interesting question. Moreover, as shown in Figure 1G and GSEA analyses, how these genes function together to promote lung cancer development remains another question of interest.

In this study, we did not measure the level of FGF19 concurrently. As shown in our study, as long as KLB was overexpressed, tumor inhibition was observed regardless of the level of FGF19 in the cancer cells. This result suggests that KLB might also serve as a molecular switch in the FGF19/FGFR4 signaling axis. It was reported that KLB in partnership with FGFR4 induced apoptosis and inhibited tumor cell proliferation in liver cancer, which was correlated with depression of the AKT and mTOR pathways 22. Our results showed that overexpression of KLB promoted apoptosis and down-regulated BCL2. Overexpression of KLB was also correlated with depression of the ERK, STAT3 and AKT pathways. STAT3 was reported to be a potential immunotherapy biomarker in oncogene-addicted NSCLC and elucidation of the role of KLB in NSCLC metastases via regulation of STAT3 should be further investigated 41, 42.

FGF21, another ligand of KLB, was reported to extend lifespan in mice by blunting IGF1 signaling pathway in the liver 17. KLB is required for metabolic activity of FGF21 18 as FGF21 binds KLB with high affinity (K_d_ value = 43.5 nM) 19, while KLB and FGFR1 have a relatively low binding affinity (K_d_ value = 1 μM). FGFR1 was also highly expressed in NSCLC and promoted tumor formation in lung cancer by interacting with SOX2, Hippo/YAP1 or Hedgehog pathway as our previously work reported 12, 13, 20, while deficiency of KLB did not influence the FGFR1 levels in cultured endothelial cells 21. This may explain the phenomenon that high expression of FGFR1 and low expression of KLB in lung cancer tissues were detected. Thus, KLB also act as a co-receptor of FGFR1 and function as the physiological receptors for FGF21.

GSEA analysis revealed that KLB up genes were involved in p53 and PPAR signaling pathway. TP53, a canonical tumor suppressor, has preeminent importance in regulating cell proliferation 43. In lung cancer, PPARγ inhibits development of primary tumors and metastases in lung cancer, and activation of PPARγ can promote apoptosis 44. Thus, the anti-tumorigenic role of KLB might be governed through the activation of P53 and PPAR. Further researches are need to understand the mechanism of KLB in lung cancer. Furthermore, MYC, as a driver of susceptibility to Aurora kinase inhibition in small cell lung cancer cells and tumors 45, was enriched in KLB down genes. It was also reported that MYC could enhance the oncogenic effect of FGFR1 in squamous cell lung cancer. This might also be an interesting point for further study to verify the relationship of MYC and KLB and explore the combined treatment of NSCLC.

In conclusion, the present work has evaluated the diagnostic/prognostic/therapeutic value of KLB in NSCLC, and it could be a target for theranostics in NSCLC.

## Supplementary Material

Supplementary figures and tables.

## Abbreviations

NSCLC: non-small cell lung cancer
KLB: βKlotho
LSQ: lung squamous carcinoma
LADC: lung adenocarcinoma
LLC: lung large cell carcinoma
qRT-PCR: Quantitative reverse transcription polymerase chain reaction
GSEA: gene set enrichment analysis
IHC: immunohistochemistry
IF: immunofluorescence microscopy
KLB-OE: βKlotho overexpressing vector
KLB-EV: βKlotho empty vector
OS: overall survival
PFS: progression-free survival

## References

[1] Siegel RL, Miller KD, Jemal A (2019). Cancer Statistics, 2019. CA Cancer J Clin.

[2] Chen WQ, Zheng RS, Baade PD, Zhang SW, Zeng HM, Bray F (2016). Cancer Statistics in China, 2015. CA Cancer J Clin.

[3] Yoda S, Dagogo-Jack I, Hata AN (2019). Targeting Oncogenic Drivers in Lung Cancer: Recent Progress, Current Challenges and Future Opportunities. Pharmacol Ther.

[4] Razzaque MS (2012). The Role of Klotho in Energy Metabolism. Nat Rev Endocrinol.

[5] Sahu A, Mamiya H, Shinde SN, Cheikhi A, Winter LL, Vo NV (2018). Age-related Declines in α-Klotho Drive Progenitor Cell Mitochondrial Dysfunction and Impaired Muscle Regeneration. Nat Commun.

[6] Zeng CY, Yang TT, Zhou HJ, Zhao Y, Kuang X, Duan W (2019). Lentiviral Vector-Mediated Overexpression of Klotho in the Brain Improves Alzheimer's Disease-Like Pathology and Cognitive Deficits in Mice. Neurobiol Aging.

[7] Kuro-o M, Matsumura Y, Aizawa H, Kawaguchi H, Suga T, Utsugi T (1997). Mutation of the Mouse Klotho Gene Leads to a Syndrome Resembling Ageing. Nature.

[8] Dubal DB, Zhu L, Sanchez PE, Worden K, Broestl L, Johnson E (2015). Life Extension Factor Klotho Prevents Mortality and Enhances Cognition in hAPP Transgenic Mice. J Neurosci.

[9] Wolf I, Levanon-Cohen S, Bose S, Ligumsky H, Sredni B, Kanety H (2008). Klotho: A Tumor Suppressor and a Modulator of the IGF-1 and FGF Pathways in Human Breast Cancer. Oncogene.

[10] Lee J, Jeong DJ, Kim J, Lee S, Park JH, Chang B (2010). The Anti-aging Gene KLOTHO is a Novel Target for Epigenetic Silencing in Human Cervical Carcinoma. Mol Cancer.

[11] Abramovitz L, Rubinek T, Ligumsky H, Bose S, Barshack I, Avivi C (2011). KL1 Internal Repeat Mediates Klotho Tumor Suppressor Activities and Inhibits bFGF and IGF-I Signaling in Pancreatic Cancer. Clin Cancer Res.

[12] Doi S, Zou Y, Togao O, Pastor JV, John GB, Wang L (2011). Klotho Inhibits Transforming Growth Factor-Beta1 (TGF-Beta1) Signaling and Suppresses Renal FiBrosis and Cancer Metastasis in Mice. J Biol Chem.

[13] Lojkin I, Rubinek T, Orsulic S, Schwarzmann O, Karlan BY, Bose S (2015). Reduced Expression and Growth Inhibitory Activity of the Aging Suppressor Klotho in Epithelial Ovarian Cancer. Cancer Lett.

[14] Zhou L, Li Y, Zhou D, Tan RJ, Liu Y (2013). Loss of Klotho Contributes to Kidney Injury by Derepression of Wnt/beta-Catenin Signaling. J Am Soc Nephrol.

[15] Usuda J, Ichinose S, Ishizumi T, Ohtani K, Inoue T, Saji H (2011). Klotho Predicts Good Clinical Outcome in Patients with Limited-Disease Small Cell Lung Cancer Who Received Surgery. Lung Cancer.

[16] Ito S, Kinoshita S, Shiraishi N, Nakagawa S, Sekine S (2000). Molecular Cloning and Expression Analyses of Mouse Betaklotho, Which Encodes a Novel Klotho Family Protein. Mech Dev.

[17] Lee S, Choi J, Mohanty J, Sousa LP, Tome F, Pardon E (2018). Structures of β-klotho Reveal a 'zip code'-like Mechanism for Endocrine FGF Signalling. Nature.

[18] Chen G, Liu Y, Goetz R, Fu L, Jayaraman S, Hu MC (2018). α-Klotho is a non-enzymatic Molecular Scaffold for FGF23 Hormone Signalling. Nature.

[19] Hori S, Miyake M, Onishi S, Tatsumi Y, Morizawa Y, Nakai Y (2016). Clinical Significance of α- and β-Klotho in Urothelial Carcinoma of the Bladder. Oncol Rep.

[20] Poh W, Wong W, Ong H, Aung MO, Lim SG, Chua BT (2012). Klotho-beta Overexpression as a Novel Target for Suppressing Proliferation and Fibroblast Growth Factor Receptor-4 Signaling in Hepatocellular Carcinoma. Mol Cancer.

[21] Lin ZZ, Hsu C, Jeng YM, Hu FC, Pan HW, Wu YM (2019). Klotho-Beta and Fibroblast Growth Factor 19 Expression Correlates with Early Recurrence of Resectable Hepatocellular Carcinoma. Liver Int.

[22] Luo Y, Yang C, Lu W, Xie R, Jin C, Huang P (2010). Metabolic Regulator βKlotho Interacts with Fibroblast Growth Factor Receptor 4 (FGFR4) to Induce Apoptosis and Inhibit Tumor Cell Proliferation. J Biol Chem.

[23] Ye X, Guo Y, Zhang Q, Chen W, Hua X, Liu W (2013). Betaklotho Suppresses Tumor Growth in Hepatocellular Carcinoma by Regulating Akt/GSK-3beta/Cyclin D1 Signaling Pathway. PLoS One.

[24] Lee KJ, Jang YO, Cha SK, Kim MY, Park KS, Eom YW (2018). Expression of Fibroblast Growth Factor 21 and β-Klotho Regulates Hepatic Fibrosis through the Nuclear Factor-κB and c-Jun N-Terminal Kinase Pathways. Gut Liver.

[25] Ji W, Yu Y, Li Z, Wang G, Li F, Xia W (2016). FGFR1 Promotes the Stem Cell-Like Phenotype of FGFR1-Amplified Non-Small Cell Lung Cancer Cells through the Hedgehog Pathway. Oncotarget.

[26] Gyorffy B, Surowiak P, Budczies J, Lanczky A (2014). Online Survival Analysis Software to Assess the Prognostic Value of Biomarkers Using Transcriptomic Data in Non-Small-Cell Lung Cancer. PLoS One.

[27] Subramanian A, Tamayo P, Mootha VK, Mukherjee S, Ebert BL, Gillette MA (2005). Gene Set Enrichment Analysis: a Knowledge-Based Approach for Interpreting Genome-Wide Expression Profiles. Proc Natl Acad Sci U S A.

[28] Wang K, Ji W, Yu Y, Li Z, Niu X, Xia W (2018). FGFR1-ERK1/2-SOX2 axis promotes cell proliferation, epithelial-mesenchymal transition, and metastasis in FGFR1-amplified lung cancer. Oncogene.

[29] Tan Q, Li F, Wang G, Xia W, Li Z, Niu X (2016). Identification of FGF19 as a Prognostic Marker and Potential Driver Gene of Lung Squamous Cell Carcinomas in Chinese Smoking Patients. Oncotarget.

[30] Kwon OS, Hong SK, Kwon SJ, Go YH, Oh E, Cha HJ (2017). BCL2 Induced By LAMTOR3/MAPK is a Druggable Target of Chemoradioresistance in Mesenchymal Lung Cancer. Cancer Lett.

[31] Wang Y, Chen L, Huang G, He D, He J, Xu W (2013). Klotho Sensitizes Human Lung Cancer Cell Line to Cisplatin via PI3k/Akt Pathway. PLoS One.

[32] Mencke R, Olauson H, Hillebrands JL (2017). Effects of Klotho on Fibrosis and Cancer: A Renal Focus on Mechanisms and Therapeutic Strategies. Adv Drug Deliv Rev.

[33] Cooper GM (2000). The Cell: A Molecular Approach. 2nd ed.

[34] Prabhu VV, Devaraj SN (2017). KAI1/CD82, Metastasis Suppressor Gene as a Therapeutic Target for Non-Small-Cell Lung Carcinoma. J Environ Pathol Toxicol Oncol.

[35] Zhang ZL, Wang NN, Ma QL, Chen Y, Yao L, Zhang L (2019). Somatic and germline mutations in the tumor suppressor gene PARK2 impair PINK1/Parkin-mediated mitophagy in lung cancer cells. Acta Pharmacol Sin.

[36] Chen KY, Chen CC, Chang YC, Chang MC (2019). Resveratrol induced premature senescence and inhibited epithelial-mesenchymal transition of cancer cells via induction of tumor suppressor Rad9. PLoS One.

[37] Yang Y, Ding L, Hu Q, Xia J, Sun J, Wang X (2017). MicroRNA-218 functions as a tumor suppressor in lung cancer by targeting IL-6/STAT3 and negatively correlates with poor prognosis. Mol Cancer.

[38] Inamura K (2017). Major Tumor Suppressor and Oncogenic Non-Coding RNAs: Clinical Relevance in Lung Cancer. Cells.

[39] Lin BC, Wang M, Blackmore C, Desnoyers LR (2007). Liver-specific Activities of FGF19 Require Klotho Beta. J Biol Chem.

[40] Wu X, Ge H, Lemon B, Weiszmann J, Gupte J, Hawkins N (2009). Selective Activation of FGFR4 by an FGF19 Variant does not Improve Glucose Metabolism in ob/ob Mice. Proc Natl Acad Sci U S A.

[41] Attili I, Karachaliou N, Bonanno L, Berenguer J, Bracht J, Codony-Servat J (2018). STAT3 as a Potential Immunotherapy Biomarker in Oncogene-Addicted Non-Small Cell Lung Cancer. Ther Adv Med Oncol.

[42] Baek JH, Yun HS, Kwon GT, Kim JY, Lee CW, Song JY (2018). PLOD3 Promotes Lung Metastasis via Regulation of STAT3. Cell Death Dis.

[43] Hanahan D, Weinberg RA (2011). Hallmarks of Cancer: The Next Generation. Cell.

[44] Reddy AT, Lakshmi SP, Reddy RC (2016). PPARγ as a Novel Therapeutic Target in Lung Cancer. PPAR Res.

[45] Brägelmann J, Böhm S, Guthrie MR, Mollaoglu G, Oliver TG, Sos ML (2017). Family Matters: How MYC Family Oncogenes Impact Small Cell Lung Cancer. Cell Cycle.

