# Erratum: βKlotho is identified as a target for theranostics in non-small cell lung cancer: Erratum

**DOI:** 10.7150/thno.46029

**Published:** 2020-04-12

**Authors:** Fan Li, Xiyao Li, Ziming Li, Wenxiang Ji, Shun Lu, Weiliang Xia

**Affiliations:** 1 State Key Laboratory of Oncogenes and Related Genes, Renji-Med X Clinical Stem Cell Research Center, Ren Ji Hospital, School of Medicine and School of Biomedical Engineering, Shanghai Jiao Tong University, Shanghai, China; 2 Shanghai Lung Cancer Center, Shanghai Chest Hospital, Shanghai Jiao Tong University, Shanghai, China

In our paper 1, Figure 3 should be corrected as follows.

The corrected figures do not affect the original conclusions of the findings.

## Figures and Tables

**Figure 3:**
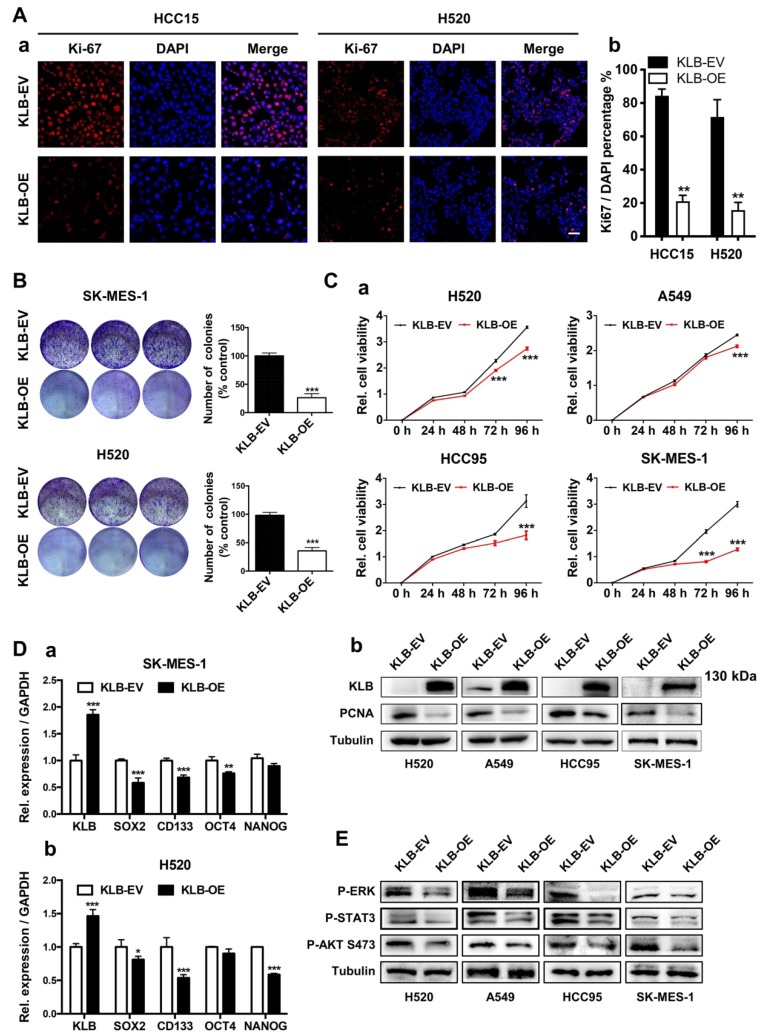
** High levels of KLB inhibit the proliferation and inhibits ERK, STAT3, AKT pathway of human NSCLC cells. A.** Cancer cells were transfected with a pCDH-KLB expression vector (KLB-OE) or a control pCDH vector (KLB-EV). IF staining of Ki67(red) and DAPI (for nucleus, blue) in HCC15 and H520 cells after transfection. Scale bar = 50 μm. The right panel was % of Ki-67 positive cells. **B.** Colony formation in SK-MES-1 and H520 cells. **C. (a)** Cell viability assay of stably overexpressed (KLB-OE) and control (KLB-EV) cancer cells. **(b)** Protein levels of KLB and proliferation marker PCNA were determined by western blot. **D.** qRT-PCR analysis of stemness-related genes in **(a)** SK-MES-1 cells and **(b)** H520 cells. E. Overexpression of KLB suppressed ERK, STAT3, AKT pathway. Data were represented as mean and SEM from three independent experiments. * P < 0.05. ** P < 0.01. *** P < 0.001.
