# βKlotho is identified as a target for theranostics in non-small cell lung cancer: Erratum

**DOI:** 10.7150/thno.86780

**Published:** 2023-08-06

**Authors:** Fan Li, Xiyao Li, Ziming Li, Wenxiang Ji, Shun Lu, Weiliang Xia

**Affiliations:** 1 State Key Laboratory of Oncogenes and Related Genes, Renji-Med X Clinical Stem Cell Research Center, Ren Ji Hospital, School of Medicine and School of Biomedical Engineering, Shanghai Jiao Tong University, Shanghai, China; 2 Shanghai Lung Cancer Center, Shanghai Chest Hospital, Shanghai Jiao Tong University, Shanghai, China

The second Tubulin band in Figure 1A a was inadvertently misplaced. The correct figures are shown below in Figure 1A a. We conducted the quantitative analysis of the bands, as shown in new Figure 1A b.

The corrected figures do not affect the original conclusions of the findings. The authors sincerely apologize for any inconvenience these errors may have caused.

## Figures and Tables

**Figure 1:**
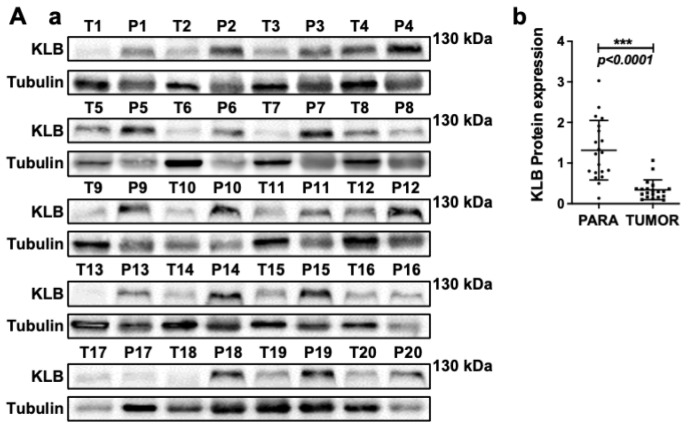
Correct Figure 1A.

