# Supplementary material for: βKlotho is identified as a target for theranostics in non-small cell lung cancer
Source: Theranostics. 2019 Oct 12;9(25):7474–89. doi: 10.7150/thno.35582 (PMC6831461; doi:10.7150/thno.35582)
Supplement: Supplementary file 1 — Supplementary figures and tables. [file thnov09p7474s1.pdf]

Supplementary Figures

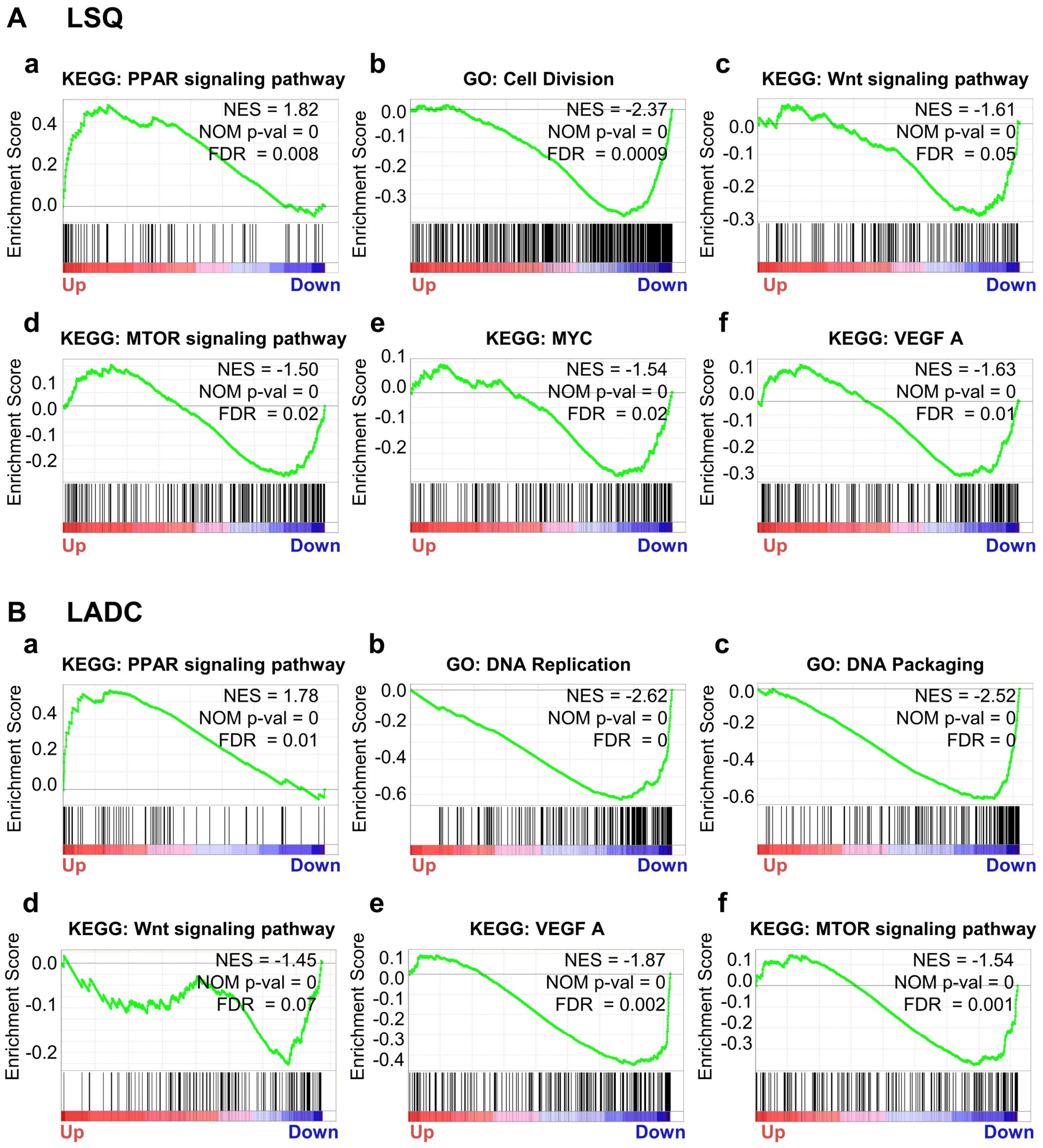

Supplementary Figure 1. Gene set enrichment analysis plots for KLB in Squamous cell lung cancer and in Adenocarcinoma. A. In squamous cell lung cancer, KLB up-regulated genes were involved in **(a)** PPAR signaling pathway and down-regulated genes were involved in **(b)** cell division, **(c)** WNT, **(d)** MTOR, **(e)**

MYC and **(f)** VEGF_A signaling. **B.** In adenocarcinoma, KLB up-regulated genes were involved in **(a)** PPAR signaling pathway and down-regulated genes were involved in **(b)** DNA replication **(c)** DNA packaging, **(d)** WNT, **(e)** VEGF-A and **(f)** MTOR signaling.

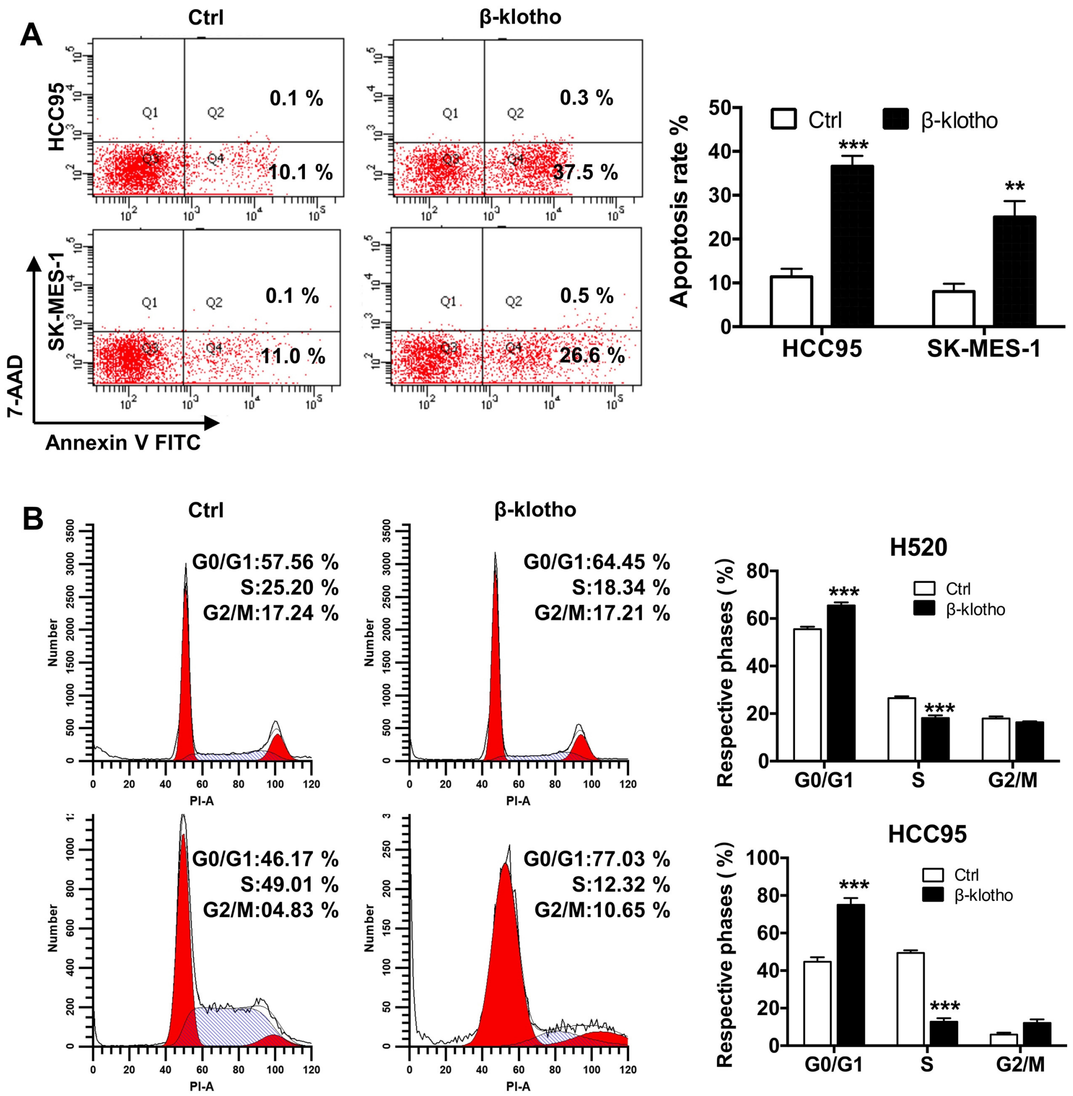

Supplementary Figure 2. Exogenous βKlotho promoted apoptosis and arrests cell cycle. A. Exogenous βKlotho induced apoptosis. βKlotho was added to cultures

HCC95 and SK-MES-1 cells. After 72 h, FACS- based Annexin V/7-AAD assays were conducted to determine apoptosis. Right panel: quantifications of the results from the FACS-based study. **B.** Representative histograms depicting cell cycle profiles of H520 cells and HCC95 cells cultured with exogenous KLB for 72 h. Right panel: Quantifications of the histograms Data were collected from three independent experiments. *$p < 0.05$. ** $P < 0.01$. ***$P < 0.001$.

Supplementary Table 1. Clinicopathological characteristics of KLB expression

Clinical pathological features	SCC			ADC			Serum		
	high N = 7	Low N = 13	*P* value	high N = 10	Low N = 20	*P* value	high N = 34	Low N = 23	*P* value
Age (years)			0.274			0.729			0.640
< 50	0(0.0)	2(15.4)		2(20.0)	3(15.0)		6(17.6)	3(13.0)	
≥ 50	7(100.0)	11(84.6)		8(80.0)	17(85.0)		28(82.4)	20(87.0)	
Gender			0.639			0.584			0.423
Male	6(85.7)	12(92.3)		4(40.0)	6(30.0)		25(73.5)	19(82.6)	
Female	1(14.3)	1(7.7)		6(60.0)	14(70.0)		9(26.5)	4(17.4)	
Tumor size (cm)			0.639			0.602			0.205
< 5	4(57.1)	6(42.9)		5(50.0)	8(40.0)		22(64.7)	11(47.8)	
≥ 5	3(42.9)	7(57.1)		5(50.0)	12(60.0)		12(35.5)	12(52.2)	
Smoking status			0.052			0.76			0.298
yes	4(57.1)	2(21.4)		2(20.0)	5(25.0)		21(61.8)	11(47.8)	
no	3(42.9)	11(78.6)		8(80.0)	15(75.0)		13(38.2)	12(52.2)	
N stage			**0.043**			0.07			0.135
N < 2	6(85.7)	5(35.7)		7(70.0)	7(35.0)		11(32.4)	12(52.2)	
N ≥ 2	1(14.3)	8(64.3)		3(30.0)	13(65.0)		23(67.6)	11(47.8)	

Values are given as No. (%), unless otherwise indicated. Categorical variables were compared by using the Chi square test or Fisher's exact test, and statistical significance is shown in bold ($P < 0.05$), SCC: squamous cell carcinoma, ADC: adenocarcinoma.

Supplementary Table 2. Real-time PCR primers

GENE	FORWARD PRIMERS （5'～3'）	REVERSE PRIMERS （5'～3'）
KLB	GCCATCATCGCACAAGAATCC	CTTACCTGTTTGCTCCTTTCAAGAG
CyclinD1	GCTGCGAAGTGGAAACCATC	CCTCCTTCTGCACACATTTGAA
SOX2	GTATCAGGAGTTGTCAAGGC	AGTCCTAGTCTTAAAGAGG
CD133	TCCACAGAAATTTACCTACATTGG	CAGCAGAGAGCAGATGACCA
OCT4	GCAATTTGCCAAGCTCCTGAA	GCAGATGGTCGTTTGGCTGA
Nanog	CCTGTGATTTGTGGGCCTG	GACAGTCTCCGTGTGAGGCAT
E-cadherin	CGAGAGCTACACGTTCACGG	GGGTGTCGAGGGAAAAATAGG
N-cadherin	TTTGATGGAGGTCTCCTAACACC	ACGTTTAACACGTTGGAAATGTG
Vimentin	GACGCCATCAACACCGAGTT	GACGCCATCAACACCGAGTT
Snail	TCGGAAGCCTAACTACAGCGA	AGATGAGCATTGGCAGCGAG
GAPDH	GGAGCGAGATCCCTCCAAAAT	GGCTGTTGTCATACTTCTCATGG
